# Identification of Metabolites of the Dietary Supplement Ingredient 5α‐Hydroxy‐Laxogenin

**DOI:** 10.1002/dta.70038

**Published:** 2026-02-17

**Authors:** Daniel Derwand, Oliver Zierau, Detlef Thieme, Annekathrin Martina Keiler

**Affiliations:** ^1^ Institute of Doping Analysis and Sports Biochemistry Dresden Kreischa Germany; ^2^ Environmental Monitoring & Endocrinology Faculty of Biology, Technische Universität Dresden Dresden Germany

**Keywords:** doping, hair analysis, HepG2 cells

## Abstract

Illegally added, undeclared dietary supplement ingredients represent one way of unintentional exposure to substances that are prohibited by the World Anti‐Doping Agency (WADA). In addition to the risk of anti‐doping rule violation, adulterated dietary supplements can also be a health risk for elite and recreational athletes. 5α‐hydroxy‐laxogenin is one of those illegal substances but is not prohibited yet. Though an in vitro bioassay showed androgen receptor activation, this androgenic effect could not be confirmed in the preclinical orchiectomized rat model, which might be due to a fast biotransformation into inactive metabolites. In the present study, we investigated the metabolism of 5α‐hydroxy‐laxogenin using three different approaches: in silico prediction using the GLORYx tool of NERRD, in vitro biotransformation using the human hepatocellular cell line HepG2 and the preclinical orchiectomized rat model, from which serum and hair samples were collected and investigated. Analyses were performed using high‐performance liquid chromatography‐high‐resolution mass spectrometry (HPLC‐HRMS). In silico metabolism predicted five different metabolites resulting from hydroxylation, reduction, or oxidation. The human HepG2 cells generated five metabolites resulting from reduction or hydroxylation and from a combination of both. Three metabolites generated in vitro and an additional mono‐hydroxylated metabolite were detected in the serum of the rats treated daily subcutaneously with 5α‐hydroxy‐laxogenin for 2 weeks. Hair analysis points to incorporation of 5α‐hydroxy‐laxogenin, though showing a rather high contamination most likely by saliva or urine. In conclusion, we identified and characterized metabolites of 5α‐hydroxy‐laxogenin, for which, however, the exact modification sites need to be investigated in future.

## Graphical abstract

The biotransformation of the dietary supplement ingredient 5α‐hydroxy‐laxogenin was investigated using human HepG2 cells, the rat and in silico using the online software Next‐gen E‐Resource for Drug Discovery (NERDD). We identified five metabolites generated by the human HepG2 cells of which three and an additional mono‐hydroxylated metabolite were also found in the rat serum samples. The in silico prediction yielded five metabolites. Four of those metabolites most likely coincides with the in metabolites detected in vitro and in vivo respectively and might be suitable as targets for 5α‐hydroxy‐laxogenin screening in serum.

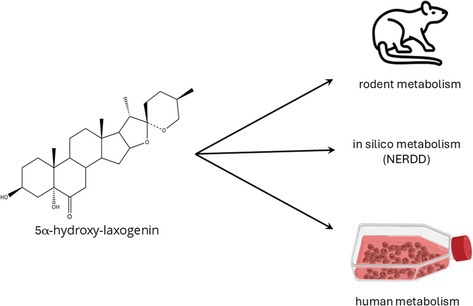

## 1 Introduction

Illegally added, undeclared dietary supplement ingredients represent one way of unintentional exposure to substances that are prohibited by the World Anti‐Doping Agency (WADA) for elite athletes [1, 2]. Beside pharmaceuticals (e.g., diuretics, androgens, and stimulants), animal‐derived traditional medicine formulations, botanical extracts, or unapproved substances have been observed as unlawfully added ingredients [3, 4, 5]. In addition to the risk of anti‐doping rule violation, adulterated dietary supplements can also be a health risk for elite and recreational athletes. 5α‐hydroxy‐laxogenin is one of those illegal substances listed in the US Food and Drug Administration's directory to warn consumers [6]. In contrast to the spirostane‐type plant steroid laxogenin, 5α‐hydroxy‐laxogenin is not a natural compound [7]. To characterize this dietary supplement ingredient in terms of potential performance enhancing effects or health risks, we recently performed in vitro and in vivo bioassays. Binding and activation of the androgen receptor have been shown using a reporter gene assay in human PC3 cells and by in silico modelling the binding of 5α‐hydroxy‐laxogenin to the receptor [8, 9]. However, this androgenic effect could not be confirmed in the preclinical orchiectomized rat model investigating 5α‐hydroxy‐laxogenin's effects on different androgen‐responsive target tissues like prostate, seminal vesicle, or the *Musculus levator ani* [9]. At present, 5α‐hydroxy‐laxogenin does not fulfil two out of three criteria defined by WADA for prohibition, which are 1. potential performance enhancement, 2. (potential) health risk for athletes, and 3. violation of the spirit of sport [1]. As biotransformation and thus potential inactivation of 5α‐hydroxy‐laxogenin might be a reason for the different outcome of the in vitro and in vivo assays, the present study aimed at the identification of metabolites. Although the performance‐enhancing effects in humans are unclear so far, the characterization of metabolites is of interest for potential future anti‐doping analyses; complement data on athletic performance improvement and harmful effects on athletes' health might be available. We herein compared the metabolism of 5α‐hydroxy‐laxogenin using human HepG2 cells and the preclinical orchiectomized rat model. The freeware tool GLORYx was further used for in silico prediction to assist metabolite identification. Moreover, we examined the potential incorporation of 5α‐hydroxy‐laxogenin in the hair matrix of rats that have been treated with 5α‐hydroxy‐laxogenin for 2 weeks. Though hair is not a matrix currently approved by WADA for anti‐doping analyses, the knowledge on the incorporation behaviour in hair can be of interest to investigate a potential substance intake retrospectively. The analysis of different hair segments allows for a retrospective estimation of the intake period. For this purpose, we performed high‐performance liquid chromatography–high‐resolution mass spectrometry analyses (HPLC‐HRMS).

## 2 Materials and Methods

### 2.1 Materials

5α‐hydroxy‐laxogenin (purity ≥ 98. %) was purchased from ChemScene (Monmouth Junction, NJ, US). Ammonium acetate and acetic acid (> 98%) were purchased from Merck (Darmstadt, Germany). Acetonitrile (ACN) and methanol (MeOH, both LC–MS grade) were supplied by Th. Geyer (Berlin, Germany). Zinc trifluoroacetate hydrate (purity > 97%), t‐butylmethylether (MBTE, 99.8%), dimethyl sulfoxide (DMSO), and castor oil were purchased from Sigma Aldrich (Taufkirchen, Germany). The MassChrom Steroids Internal Standard Mix was provided by Chromsystems (Munich, Germany). 19‐d3‐testosterone and D3‐testosterone‐glucuronide were supplied by the National Measurement Institute (Pymble, Australia). Oasis HLB SPE cartridges (1 cm^3^, 30 mg) were provided by Waters (Milford, USA). β‐Glucuronidase (from *Escherichia coli* K 12) was purchased from Roche (Mannheim, Germany). Sodium bicarbonate (NaHCO_3_, reagent grade) and potassium dihydrogen phosphate (KH_2_PO_4_, reagent grade) were obtained by KMF (Lohmar, Germany), and potassium carbonate (K_2_CO_3_, reagent grade) by Carl Roth (Karlsruhe, Germany). Sodium hydrogen phosphate (Na_2_HPO_4_, reagent grade) was purchased from Serva (Heidelberg, Germany). HepG2 cells (ACC180) were purchased from the German Collection of Microorganisms and Cell Cultures (DSZM) (Braunschweig, Germany). RPMI‐1640 medium was obtained from Sigma‐Aldrich, and fetal bovine serum (FBS) and penicillin–streptomycin (100×) were supplied by Biowest (Nuaillé, France).

### 2.2 In Silico Predictions

For in silico predictions of metabolites, the online software tool GloryX of Next‐gen E‐Resource for Drug Discovery (NERDD) was used (nerdd.univie.ac.at) [10]. For phase I metabolism, the simulation was performed including three reaction steps; hence, the possibility of three consecutive phase I reactions was allowed.

### 2.3 Animal Experiment

Handling and treatment of the male rats has been described elsewhere [9]. Animals (housed in groups of three to four animals per cage) were treated with either 1 mg/kg bw, 12 mg/kg bw or 36 mg/kg bw 5α‐hydroxy‐laxogenin by daily subcutaneous (s.c.) injection over a period of 14 days (dosage: mg/kg bw: mg/kg bodyweight). 5α‐hydroxy‐laxogenin was dissolved in DMSO and castor oil. Serum samples were collected by tail vein punctuation before treatment (d0) and at day 3 and day 10 of the treatment (100 μL per time point). At necropsy, serum samples (à 200 μL) were collected by heart punctuation. All serum samples were stored at −18°C. Hair samples were collected by shaving the back of the rats at the time of necropsy.

### 2.4 Rat Sample Preparation

Sample preparation was carried out by the method according to König et al. 2022 [11]. We added 25 μL 124 mM zinc trifluoroacetate containing the MassChrom Internal Standard Mix for steroids (including androstenedione‐^13^C_3_, testosterone‐d_3_, cortisone‐d_8_, and cortisol‐d_4_) to 50 μL serum sample for precipitation. After thoroughly mixing, the samples were centrifuged at 10000 rpm for 5 min. The supernatants were directly subjected to HPLC‐HRMS analysis as described below.

Hair samples from eight animals per group were extracted. Per animal, 20 mg hair were washed once with 1 mL 30% MeOH (in H_2_O) followed by centrifugation and drying at 55°C and 80 mbar. Extraction was done after addition of 1 mL MeOH (containing 1 ng/mL 19‐d_3_‐testosterone as internal standard) by homogenization using the FastPrep (MP Biomedicals, Thermo Fisher Scientific) followed by incubation in an ultrasonic bath at 60°C for 4 h. After centrifugation, the supernatant was evaporated to dryness and resolved in 15 μL ACN and 35 μL solvent A (ACN/H_2_0, 5/95 v/v with 2 mmol/L ammonium acetate and 0.1% acetic acid).

To investigate a potential contamination of the fur by the administration route or by saliva due to grooming, we performed a consecutive washing cascade of two hair samples (each 20 mg): one sample from a control animal (without 5α‐hydroxy‐laxogenin administration) and one sample from an animal treated daily with 36 mg/kg bw 5α‐hydroxy‐laxogenin for 14 days. The hair samples were washed 10 times in a row with 1 mL 30% MeOH respectively. As described above, 1 mL MeOH containing 1 ng/mL 19‐d3‐testosterone was added to each washing solution, evaporated to dryness and reconstituted in 10 μL ACN and 35 μL solvent A.

### 2.5 HepG2 Incubation

HepG2 cells were cultivated as described previously [12]. Briefly, HepG2 cells cultivated as a monolayer were incubated with 10^−6^ M 5α‐hydroxy‐laxogenin dissolved in DMSO. Medium samples (à 1 mL) were collected at 0, 24, 48, and 144 h of incubation and stored frozen at −20°C. Cell supernatants were extracted as described previously using solid phase extraction (Waters Oasis HLB cartridges 1 cm^3^, 30 mg) followed by hydrolysis with β‐glucuronidase and liquid–liquid extract with t‐butylmethylether after pH adjustment with solid carbonate buffer (NaHCO_3_ + K_2_CO_3_, 84:138, m/m) [12]. As an internal standard, D3‐testosterone‐glucuronide was added to the supernatants prior to solid‐phase extraction.

### 2.6 HPLC‐HRMS

For chromatographic separation, we used an Agilent 1290 Infinity II LC system (Agilent Technologies, Böblingen, Germany) with a Zorbax Eclipse XDB‐C8 (2.1 × 100 mm, 3.5 μm; Agilent Technologies) and the following gradient: 0 min 10% solvent B with a flow rate of 250 μL/min, 1 min 10% solvent B, 9 min 90% solvent B, 9.9 min 90% solvent B (flow rate from 1 to 9.9 min was set to 400 μL/min) and 10 min 10% solvent B with a flow rate of 250 μL/min. The injection volume of the samples was 5 μL. Solvent B consisted of ACN/H_2_0, 5/95 (v/v) with 2 mmol/L ammonium acetate and 0.1% acetic acid. The LC system was coupled to Sciex Triple TOF 6600 system (Sciex, Darmstadt, Germany). The ion source temperature was set to 500°C, the ion spray voltage was 5500 V for positive ionization, with the curtain gas pressure set to 40 psi. Ion source gas pressure 1 and 2 were set to 65 psi. For the information dependent acquisition for the serum samples, the precursor ion range was 400–700 Da with the selection fragmentation range of 150–600 Da and a collision energy of 35 eV and collision energy spread of 25 eV. For the hair sample analysis, products of 447.1 Da and 292.1 Da (d3‐testosterone), collision energy was set to 35 eV and collision energy spread was 25 eV. The analysis was achieved using Analyst TF 1.7.1, MultiQuant 3.0.3 and Peak View 2.2 software (Sciex). The calibration range for the hair analysis in the range from 0.5 to 1000 pg./mg was found to be linear from 10 to 1000 pg./mg. The detection limit was 1 pg./mg.

## 3 Results and Discussion

### 3.1 In Vitro Metabolism in Human HepG2 Cells

**FIGURE 1:**
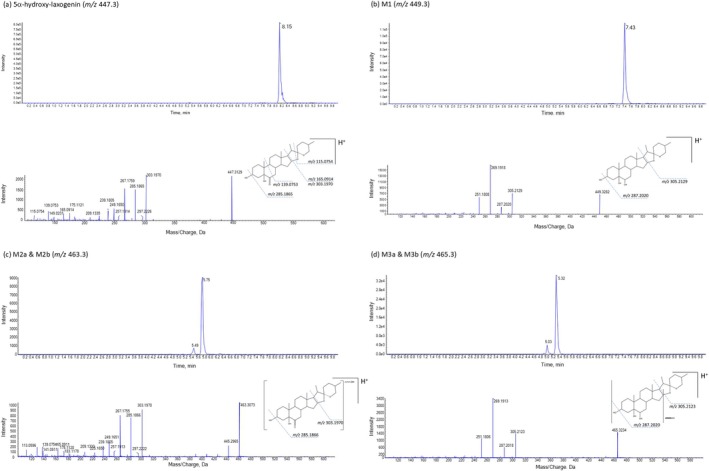
Extracted ion chromatograms and respective high‐resolution product ion mass spectra of the protonated molecules [M + H]^+^ of (a) 5α‐hydroxy‐laxogenin at *m/z* 447.3129 and the metabolites (b) **M1** at *m/z* 449.3282, (c) **M2a** and **M2b** at *m/z* 463.3073 (M2a also in rat serum; spectrum shown from M2b at 5.75 min), and (d) **M3a** and **M3b** at *m/z* 465.3234 (spectrum shown from M3b at 5.32 min) of HepG2 supernatants after 144 h of incubation.

The 5α‐hydroxy‐laxogenin molecule yielded a [M + H]^+^ molecular ion with *m/z* 447.30322 (Figure 1a). Dissociation of the protonated molecule yielded *m/z* 303.1970 due to the loss of the spirostane group and *m/z* 285.1865 and *m/z* 267.1759 due to the loss of one and two water molecules respectively (Figure 1a). The dissociation behaviour is in accordance with the results of Avula et al. [7]. We observed five 5α‐hydroxy‐laxogenin metabolites resulting from the human HepG2 cell incubation, for which structures were suggested based on the HPLC‐HRMS data and MS/MS spectra as no reference material was available. The protonated molecule of metabolite **M1** (*m/z* 449.3261) resulted from a reduction most probably of the keto‐group at the B‐ring (Figure 1b). The diagnostic fragment ions of **M1** were found at *m/z* 305.2129 and *m/z* 287.2020 (Table 1). The shift of the sterane ring fragments by two hydrogens (e.g., 303 → 305) indicates a reduction of the 6‐keto group. Moreover, two metabolites, **M2a** and **M2b**, were formed by the HepG2 cells by mono‐hydroxylation with **M2b** being the more abundant one (Figure 1c). The protonated molecules of **M2a** (*m/z* 463.3055) gave rise to four intense fragment ions at *m/z* 445.29675 (water loss), *m/z* 303.1979 (loss of the spirostane group), *m/z* 285.1854 and *m/z* 267.1755 (both elimination of water) (Table 1). The protonated molecule of the more abundant mono‐hydroxylated metabolite **M2b** (*m/z* 463.3075) gave also rise to four intense fragment ions at *m/z* 445.2965 (water loss), *m/z* 303.1970 (loss of the spirostane group), *m/z* 285.1866 and *m/z* 267.1755 (both elimination of water) (Figure 1d, Table 1). The observation of the characteristic sterane fragments (303, 285, and 267) in the parent compound and the **M2** metabolites indicates that the hydroxylation is located at the furane or pyran heterocycles. The exact positions of the hydroxylation cannot be identified based on our results. Two other metabolites, **M3a** and **M3b** (*m/z* 465.3210), resulted from a reduction of the keto‐group at the B‐ring and a mono‐hydroxylation, respectively, showing identical spectra (Figure 1d). The protonated molecule gave rise the fragment ion *m/z* 305.2123 by loss of the spirostane group, and to the three fragment ions *m/z* 287.2018, *m/z* 269.1913 and *m/z* 251.1806 by consecutive water loss (Figure 1d, Table 1). The observation that **M1** and **M3b** share identical product ions suggests that **M3b** most probably results from the hydroxylation of the **M1**. The exact hydroxylation site also remains unclear for **M3a** and **M3b**. The abundance of all metabolites showed a time‐dependent increase over the supernatant collection time points (24, 48, and 144 h).

**TABLE 1:** Retention times, observed product ions, ion transition in positive electrospray ionization (ESI), and elemental composition of the parent compound 5α‐hydroxy‐laxogenin and its metabolites **M1**, **M2**, and **M3**, and the internal Standard d3‐testosterone.

Compound (putative)	Product ion (*m/z*)	Modification	RT (min)	Diagnostic fragmentation (*m/z*)	Elemental composition
5α‐hydroxy‐laxogenin	447.3105		8.15	447 → 303.2 447 → 285.2 447 → 165.2	C_27_H_42_O_5_
**M1** (HepG2 & rat)	449.3261	Reduction	7.42	449.3 → 305.2 449.3 → 287.2	C_27_H_44_O_5_
**M2a** & **M2b** (HepG2) **M2c** (rat)	463.3054 & 463.3075 463.3081	Mono‐hydroxylation	HepG2: (minor 5.48) 5.75 rat: 5.62	463.3 → 445.2 463.3 → 303.2 463.3 → 285.2 463.3 → 267.2	C_27_H_42_O_6_
**M3a** & **M3b** (HepG2 & rat)	465.3210	Mono‐hydroxylation, reduction	HepG2: (minor 5.03) 5.32 rat: 5.0 (minor 5.37)	465.3 → 305.2 465.3 → 287.2 465.3 → 269.2 465.3 → 251.2	C_27_H_44_O_6_
d3‐testosterone (IS)	292.2355		6.49	292.2 → 109.0 292.2 → 100.0	C_19_H_25_D_3_O_2_

### 3.2 In Vivo Metabolism in Rats

**FIGURE 2:**
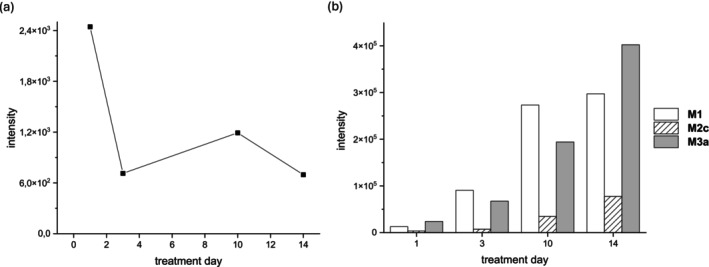
(a) Abundance of 5α‐hydroxy‐laxogenin in rat serum treated with 36 m/kg 5α‐hydroxy‐laxogenin for 1, 3, 10, or 14 days respectively. (b) Time‐dependent abundance of **M1**, **M2a,** and **M3a** in serum sample of rats treated with 36 mg/kg bw/d 5α‐hydroxy‐laxogenin for 1, 3, 10, or 14 days, respectively.

In the rodent serum samples, the parent compound was less intense compared to the metabolites. The intensity of 5α‐hydroxy‐laxogenin slowly decreased at a half‐life of approx. 11 days (Figure 2a). This seems to indicate a slow release of the compound from a depot potentially formed by the s.c. injection of 5α‐hydroxy‐laxogenin in castor oil. Regarding metabolites, we detected four metabolites in the rat serum samples, namely **M1**, **M2c**, **M3a**, and **M3b** (Table 1). The formation of the metabolites was clearly correlated with time and dosage administered, exemplarily depicted for the highest treatment dosage in Figure 2b.

### 3.3 In Silico Metabolism

Among the metabolites predicted in silico, five reached a score higher than 0.20 (Table 2). The highest score (0.46) was reached for one metabolite resulting from hydroxylation at the A ring (pM1) and one resulting from carbonyl reduction (pM2). The three other predicted metabolites (priority score of 0.22) were based on either oxidation (pM3) or mono‐hydroxylation (pM4 and pM5).

**TABLE 2:** Predicted metabolites (pMx) for 5α‐hydroxy‐laxogenin (canonical SMILES: C[C@H](CC1)CO[C@]21O[C@@]3([H])C[C@@]4([H])[C@]5([H])CC([C@@]6(O)C[C@@H](O)CC[C@]6(C)[C@@]5([H])CC[C@]4(C)[C@@]3([H])[C@@H]2C) = O) by GLORYx choosing the options “Phase I metabolites” with their rank positions (based on their priority score) and the formula. Only metabolites with a score ≥ 0.20 are depicted.

pM	Reaction type	Elemental composition	Rank	Priority score
1	Aliphatic_hydroxylation (sec_carbon_next_to_SP2, in_a_ring)	C_27_H_42_O_6_	1	0.46
2	Carbonyl_reduction_(aliphatic)	C_27_H_44_O_5_	1	0.46
3	Secondary_alcohol_oxidation_(aliphatic)	C_27_H_42_O_6_	3	0.22
4	Aliphatic_hydroxylation_(sec_carbon_in_a_ringB)	C_27_H_42_O_6_	3	0.22
5	Aliphatic hydroxylation	C_27_H_42_O_6_	3	0.22

Comparing the three models used herein to investigate the metabolism of 5α‐hydroxy‐laxogenin, the in silico modelled metabolite pM2 resulting from a reduction most likely matches **M1** being generated in the human cell line HepG2 as well as in the rat model. The two hydroxylated metabolites **M2a** and **M2b** generated in the in vitro assay were most probably the in silico predicted pM1 and pM4. Hence, the in silico approach was able to predict three of the metabolites observed in both models. Although in silico predictions can assist in metabolite identification, a definite assignment to metabolites observed in vitro or in vivo is not possible due to the lack of knowledge of the exact modification sites.

### 3.4 Hair Incorporation of 5α‐Hydroxy‐Laxogenin

Though hair is no matrix currently approved by WADA for anti‐doping analyses, the knowledge on the incorporation behaviour in hair can be of interest to investigate a potential substance intake. The analysis of different human hair segments allows for a retrospective estimation of the intake period. To investigate the incorporation behaviour of 5α‐hydroxy‐laxogenin, we analysed rat hair samples collected at 14 days of daily s.c. treatment with 36 mg/kg bw 5α‐hydroxy‐laxogenin. After the two‐week experimental time, 5α‐hydroxy‐laxogenin was observed in extracts of the hair collected at the back of the rats at necropsy after one washing step (Table 3). Only metabolite **M1** was detectable in the hair samples at significantly reduced abundance. In rats, the contamination of the matrix hair by saliva because of fur care (grooming) is very likely. Besides, urine present in the bedding is another possible contamination way. The observation of metabolite **M1** in the solution after hair washing depicts this contamination route. Hence, we performed a consecutive washing cascade of a hair sample (of an animal treated with 36 mg/kg bw) using 30% MeOH respectively. As shown in Table 4, the 5α‐hydroxy‐laxogenin concentration determined in the washing solution decreased per washing step. However, the observation that the concentration in the washing solution after the first washing step was 10‐times that quantified in the hair extract illustrates the described contamination routes.

**TABLE 3:** 5α‐hydroxy‐laxogenin concentration in hair collected at the end of the two‐week subcutaneous administration period after one washing step (*n* = 8). Animals were treated daily with either 1 mg/kg bw, 12 mg/kg bw or 36 mg/kg bw 5α‐hydroxy‐laxogenin for 14 days.

5α‐hydroxy‐laxogenin dosage [mg/kg bw]	Hair concentration [pg/mg]
1 mg/kg	324.5 ± 122.9
12 mg/kg	814.7 ± 220.1
36 mg/kg	771.9 ± 297.2

**TABLE 4:** Washing cascade of a 20 mg hair sample from one animal treated for 14 days with 36 mg/kg 5α‐hydroxy‐laxogenin. The washing solution of each step (1 ml MeOH) was evaporated, reconstituted and analysed.

Washing step	Concentration [pg/mg]
1	8850.796
2	2974.22
3	865.062
4	695.54
5	446.501
6	231.792
7	297.487
8	89.891
9	155.612
10	187.214

### 3.5 Limitations

Limitations of the present study are the lack of exact metabolic reaction sites in the 5α‐hydroxy‐laxogenin structure, which can be only determined by NMR spectroscopy elucidation of the isolated metabolites or synthesized reference materials. This also prevents a definite correlation to the structures predicted by the in silico approach. Moreover, the presence of the identified metabolites needs to be proven after human administration. In addition, the route of administration might be of interest as 5α‐hydroxy‐laxogenin containing supplements sold are administered orally, whereas rats were treated s.c. in the present study.

## 4 Conclusion

In conclusion, we identified metabolites of 5α‐hydroxy‐laxogenin, which is sold as an anabolic dietary supplement ingredient, generated in vitro using the human hepatocyte line HepG2 and in the preclinical rat model, and used in silico metabolite prediction to assist metabolite identification. We observed five metabolites resulting from hydroxylation, reduction, and a combination of both after HepG2 incubation, which were characterized for their mass spectrometric behaviour using high‐resolution mass spectrometry. Three of those and an additional mono‐hydroxylated metabolite were also observed in the rat serum samples. Three of the in silico predicted metabolites could be assigned to the ones observed in the in vitro and the rat model. Each of the metabolites was found to be suitable as a target for 5α‐hydroxy‐laxogenin screening in serum. Even if the data available so far do not justify an inclusion of 5α‐hydroxy‐laxogenin in WADA's Prohibited List, the mass spectrometric characterization of metabolites is of interest, as data on potential human performance enhancement or health risks might be shown in future studies. We further showed incorporation of 5α‐hydroxy‐laxogenin in the rat hair, which might be of interest for retrospective investigation of 5α‐hydroxy‐laxogenin intake, though hair is currently not approved for anti‐doping analysis by WADA. However, comparatively high levels in decontamination solution indicate at least partial influence by contamination.

## Conflicts of Interest

The authors declare no conflicts of interest.

## Data Availability

The data that support the findings of this study are available from the corresponding author upon reasonable request.

## References

[1] World Anti‐Doping Agency , “Prohibited List. World Anti Doping Agency,” accessed 2026‐01‐08, https://www.wada‐ama.org/en/resources/world‐anti‐doping‐code‐and‐international‐standards/prohibited‐list.

[2] A. B. M. Merlo , L. Lobigs , T. Piper , C. Champod , and N. Robinson , “Unravelling the Threat of Contamination in Elite Sports: Exploring Diverse Sources Impacting Adverse Analytical Findings and the Risk of Inadvertent Exposure to Prohibited Substances,” Forensic Science International 365 (2024): 112240, 10.1016/j.forsciint.2024.112240. 39442273

[3] V. R. Kozhuharov , K. Ivanov , and S. Ivanova , “Dietary Supplements as Source of Unintentional Doping,” BioMed Research International 2022 (2022): 8387271, 10.1155/2022/8387271. 35496041 PMC9054437

[4] C. L. Torres , F. A. G. de Oliveira , L. F. Jooris , M. C. Padilha , and H. M. G. Pereira , “The Presence of Doping Agents in Dietary Supplements: A Glimpse Into the Brazilian Situation,” Drug Testing and Analysis 16, no. 1 (2024): 38–48, 10.1002/dta.3517. 37161689

[5] J. Zapata‐Linares and G. Gervasini , “Contaminants in Dietary Supplements: Toxicity, Doping Risk, and Current Regulation,” International Journal of Sport Nutrition and Exercise Metabolism 34, no. 4 (2024): 232–241, 10.1123/ijsnem.2023-0263. 38653450

[6] H. F. Program , “Information on Select Dietary Supplement Ingredients and Other Substances,” FDA, accessed 2026‐01‐08, https://www.fda.gov/food/dietary‐supplements/information‐select‐dietary‐supplement‐ingredients‐and‐other‐substances.

[7] B. Avula , A. G. Chittiboyina , J.‐Y. Bae , et al., “The Power of Hyphenated Chromatography‐Time of Flight Mass Spectrometry for Unequivocal Identification of Spirostanes in Bodybuilding Dietary Supplements,” Journal of Pharmaceutical and Biomedical Analysis 167 (2019): 74–82, 10.1016/j.jpba.2018.12.045. 30753977

[8] C. Beer and A. M. Keiler , “Androgenic Properties of the Dietary Supplement 5α‐Hydroxy‐Laxogenin,” Archives of Toxicology 96, no. 7 (2022): 2139–2142, 10.1007/s00204-022-03283-5. 35344071 PMC9151512

[9] D. Derwand , O. Zierau , C. A. Wolf , G. Wolber , and A. M. Keiler , “Effects of the Dietary Supplement 5α‐Hydroxy‐Laxogenin in the Orchiectomized Rat Model,” Drug Testing and Analysis 17, no. 9 (2025): 1743–1749, 10.1002/dta.3881. 40038867 PMC12401628

[10] C. De Bruyn Kops , M. Šícho , A. Mazzolari , and J. Kirchmair , “GLORYx: Prediction of the Metabolites Resulting From Phase 1 and Phase 2 Biotransformations of Xenobiotics,” Chemical Research in Toxicology 34, no. 2 (2021): 286–299, 10.1021/acs.chemrestox.0c00224. 32786543 PMC7887798

[11] S. König , S. Rzeppa , D. Thieme , and A. M. Keiler , “Agreement of Steroid Profiles in Athlete Biological Passport Residues and Corresponding Serum Samples,” Drug Testing and Analysis 16, no. 8 (2024): 761–765, 10.1002/dta.3365. 36068927

[12] A. Zschiesche , Z. Chundela , D. Thieme , and A. M. Keiler , “HepG2 as Promising Cell‐Based Model for Biosynthesis of Long‐Term Metabolites: Exemplified for Metandienone,” Drug Testing and Analysis 14, no. 2 (2022): 298–306, 10.1002/dta.3184. 34705329

